# Faculty standardized patients versus traditional teaching method to improve clinical competence among traditional Chinese medicine students: a prospective randomized controlled trial

**DOI:** 10.1186/s12909-024-05779-3

**Published:** 2024-07-24

**Authors:** Meilan Huang, Han Yang, Jing Guo, Xiaoxu Fu, Wangshu Chen, Bin Li, Shan Zhou, Ting Xia, Sihan Peng, Lijuan Wen, Xiao Ma, Yi Zhang, Jinhao Zeng

**Affiliations:** 1 Hospital of Chengdu University of Traditional Chinese Medicine, No. 39 Shierqiao Road, Chengdu, 610072 China; 2 Clinical Medical School, Chengdu University of Traditional Chinese Medicine, No. 1166 Liutai Avenue, Chengdu, 611137 China; 3 Clinical Skill Center, Clinical Medical School of Chengdu University of Traditional Chinese Medicine, No. 37 Shierqiao Road, Chengdu, 610072 China; 4 School of Pharmacy, Chengdu University of Traditional Chinese Medicine, No. 1166 Liutai Avenue, Chengdu, 611137 China

**Keywords:** Occupational standardized patients, Faculty standardized patients, Clinical competence, Education of TCM

## Abstract

**Background:**

Standardized patients (SPs) simulation training models have been widely used in various fields, the study of using SPs in Traditional Chinese medicine (TCM) is still a new filed. Previous studies have demonstrated the effectiveness of occupational SP for TCM (OSP-TCM), which has an increasingly problem of high time and financial costs. The faculty SPs for TCM (FSP-TCM) simulation training model may provide a better alternative. This study aims to test and determine whether FSP-TCM simulations are more cost-effective than OSP-TCM and traditional educational models to improve the clinical competence of TCM students.

**Methods:**

This study was a single-blind, prospective, randomized controlled trial conducted between February 2023 and October 2023. The participants were randomized into FSP-TCM group, OSP-TCM group and traditionally taught group (TT group) in the ratio of 1:1:1. The duration of this training program was 12 weeks (36 credit hours). Formative and summative assessments were integrated to evaluate the effectiveness of teaching and learning. Three distinct questionnaires were utilized to collect feedback from students, SPs, and teachers at the conclusion of the course. Additionally, analysis of cost comparisons between OSP-TCM and FSP-TCM were performed in the study.

**Results:**

The study comprised a total of 90 students, with no dropouts during the research. In the formative evaluation, students assigned to both the FSP-TCM and OSP-TCM groups demonstrated higher overall scores compared to those in the TT group. Notably, their performance in “physical examination” (*P*^a^ = 0.01, *P*^b^ = 0.04, *P*^c^ = 0.93) and “comprehensive ability” (*P*^a^ = 0.01, *P*^b^ = 0.006, *P*^c^ = 0.96) significantly exceeded that of the TT group. In the summary evaluation, both SP-TCM groups students outperforms TT group in the online systematic knowledge test (*P*^a^ = 0.019, *P*^b^ = 0.04, *P*^c^ = 0.97), the application of TCM technology (*P*^a^ = 0.01, *P*^b^ = 0.03, *P*^c^ = 0.93) and real-time assessment (*P*^a^= 0.003, *P*^b^ = 0.01, *P*^c^ = 0.93). The feedback questionnaire demonstrated that both SP-TCM groups showed higher levels of agreement for this course in “satisfaction with the course” (*P*^a^ = 0.03; *P*^b^ = 0.02) and “enhanced TCM clinical skills” (*P*^a^ = 0.02; *P*^b^ = 0.03) than TT group. The SP questionnaire showed that more FSPs than OSPs in “provided professional feedback” (FSPs: strongly agree 30%, agree 50% vs. OSPs: strongly agree 20%, agree 40%. *P* = 0.69), and in “gave hints” during the course (FSPs: strongly agree 10%, agree 30% vs. OSPs: strongly agree 0%, agree 10%. *P* = 0.42). It is noteworthy that FSP-TCM was significantly lower than the OSP-TCM in overall expense (FSP-TCM $7590.00 vs. OSP-TCM $17415.60), and teachers have a positive attitude towards the FSP-TCM.

**Conclusion:**

FSP-TCM training mode showed greater effectiveness than traditional teaching method in improving clinical competence among TCM students. It was feasible, practical, and cost-effective, and may serve as an alternative method to OSP-TCM simulation.

**Supplementary Information:**

The online version contains supplementary material available at 10.1186/s12909-024-05779-3.

## Introduction

Standardized patients (SPs) are simulated patients trained based on specific standards and procedures and can simulate various diseases and individual characteristics encountered in real medical scenarios [1]. They present the clinical features of real patients in a standardized manner, including different conditions, physical signs, symptoms, and needs of patients [2]. Conventionally, occupational SPs (OSPs) were recruited from the general public, including professional actors and volunteers. These individuals were organized by relevant institutions and underwent training to provide consistent services to educational institutions, including medical schools [3].

The lack of practical experience among medical students is a common problem in the traditional model of medical education [4]. Uncertainty of clinical work poses challenges and elicits anxiety during the transition to clinical practice in most medical students [5, 6]. However, SPs have reversed this situation. The efficacy of SPs in improving students’ clinical competence, reducing pre-clinical anxiety [7], and boosting confidence before clinical practice, has been demonstrated and regarded as a useful complement to traditional clinical experiences [8]. SPs as a situated teaching model, can illustrate medical student deficits in communication skills, thinking, and diagnostic and therapeutic accuracy [9], and can assess students’ proficiency in clinical skills, facilitating the adjustment and optimization of teaching strategies [10, 11]. The diverse advantages of SPs have contributed to their popularity and wide adoption.

Traditional Chinese Medicine (TCM) is increasingly recognized as an important complementary and alternative medicine worldwide, encompassing herbal medicine, acupuncture, moxibustion, and therapeutic massage, etc. [12, 13]. TCM plays a vital role in China’s healthcare system, with the reports from the State Administration of TCM indicating that 828,871 TCM practitioners were registered nationwide by 2020, comprising 17.2% of the country’s healthcare professionals (http://www.natcm.gov.cn/). Moreover, over 800 of colleges, universities and technical schools nationwide currently established TCM programs (including 44 TCM colleges and universities, 150 western medicine colleges and universities, 250 non- medical colleges and universities, 39 TCM technical schools, 135 western medicine technical schools and 204 non-medical technical schools). TCM programs are classified as first-tier disciplines by the Ministry of Education, underscoring the Chinese government’s profound commitment to educating and nurturing TCM students. Currently, SPs are widely used in various fields, including nursing, psychology, pharmacy, and clinical training [8, 14]. However, the integration of SPs into Chinese medicine education remains an under-explored area. As an independent discipline, TCM has distinctive features [15]. For example, in terms of clinical skills, TCM emphasizes cultivating student ability of the four diagnostic methods (inspection, auscultation, inquiry, and palpation) and TCM syndrome differentiation [16]. The disciplinary characteristics differ between TCM and Western medicine, requiring specifically trained SPs of TCM.

Our previous studies have progressively introduced courses involving both OSPs, student standardized patients, and virtual standardized patients based on TCM students feedback to enhance clinical skills [12, 17–19]. These studies have demonstrated that OSP-TCM simulated training significantly improves students’ clinical abilities compared to conventional methods [18]. Nevertheless, the training and utilization of OSPs incur high time and financial costs. Consequently, ongoing efforts are focused on developing a viable, practical, and cost-effective training model. Currently, studies on faculty standardized patients (FSPs) in the field of TCM education are scarce, and the effectiveness of different models to improve students’ clinical competence remains uncertain.

A prospective, single-blind, randomized controlled trial was conducted to evaluate the clinical competency of students from FSP-TCM, OSP-TCM, and traditional teaching groups. Formative and summative assessments, questionnaires, and cost analysis were integrated to evaluate the effectiveness. This study aims to test the hypothesis that FSP-TCM, as compared to OSP-TCM and traditional teaching mode, may be a feasible, practical, and cost-effective mode of training simulation.

## Methods

### Ethics review and approval

This study was registered in the Educational Administration System of CDUTCM (The registration number: 1,130,730), and the Ethics Committee of CDUTCM has approved our study protocol (The grant number: 1,005,510). The study complied with ethical principles and regulations to fully safeguard the rights and interests of all participants [20]. Curriculum arrangements were made following the “Medical Education Standards of Undergraduate Education- Chinese Medicine” issued by the Chinese Ministry of Education and the “Five-Year Undergraduate Education Guide of TCM” issued by CDUTCM. All participants received comprehensive information before signing the informed consent form and voluntarily agreed to participate in the study.

### Trainee recruitment

The screening was performed by sophomore students majoring in TCM (five-year program) who were studying TCM clinical competency training program at CDUTCM in 2023. By reviewing relevant literature and referencing effect sizes [18, 21], then using PASS 15 software for sample size estimation, the minimum sample size was determined to be 75.

### Inclusion and exclusion criteria

The inclusion criteria were: (1): Sophomore students majoring in TCM (five-year program) studying at CDUTCM in 2023. (2):19–23 years old, male or female. (3): Voluntary confidentiality agreements that are signed and informed consent. (4): Participants had passed the examination of the basic Chinese medicine course and the basic western medicine course. (5): Physically and mentally healthy enough to complete the study.

The exclusion criteria were: (1): Participants who had previously participated in formal training for standardized patients. (2): Trainees who had violated the confidentiality agreement of the course content during the training period. (3) Trainees who were unwilling or unable to complete the training due to the trainees’ own reason.

### Randomization and blinding

SPSS 27.0 software was utilized to generate 90 random numbers, which were then randomly split into three groups, each group representing a specific intervention. Researchers assigned random numbers to eligible students, and then used these numbers to allocate the students into different groups. Randomization was conducted by an individual with no exposure to participants, ensuring confidentiality in participant allocation and baseline information throughout the study. The Data were collected and managed by individuals who was blinded to the study. Subsequently, an independent individual conducted the data analysis upon completion of data collection.

### Training and qualification of FSP-TCM and OSP-TCM

Volunteers were recruited based on predefined criteria before the start of the study who need to have been teaching in the field for 5 years or more. They underwent physical and mental health assessments and received certification of good health. Additionally, volunteers were obligated to sign confidentiality and informed consent forms and demonstrate availability for training sessions (Supplement 1). They completed a rigorous training program with three seasoned SP trainers. The program included lectures delivered by the trainers, group-based skill training, and self-directed learning, which aims to give them a better understanding and presentation of real patient signs and symptoms. Upon completing the training, two seasoned SP instructors conducted volunteer evaluations using a combination of tests and performances. 10 volunteers finally passed the eligibility assessment and were enrolled as FSP-TCM for this study. Furthermore, a cohort of volunteers for OSP-TCM was recruited, trained, and certified following the criteria. There are currently 19 established OSP-TCM practitioners from whom 10 were randomly selected to participate in this study. The training methodology and eligibility assessment criteria are provided in our previous studies [18, 19] (Supplement 2).

### Training curriculum and setting

This study was conducted within the framework of a clinical skills training course in Chinese medicine, which was undertaken during the second semester of the sophomore year. This course comprises 36 credit hours and 12 representative TCM diseases. Throughout a span of 12 weeks, participants engaged in 3 credit hours of training for a target TCM disease each week (see supplement 3). The training process for members of TT group consists of six steps. (1) Teachers delivered didactic instruction. (2) Students engaged in open discussion. (3) Students collaborated in pairs to enact doctor-patient communication role-plays. (4) Teachers provide feedback on student performance. (5) Students developed medical histories and Chinese medicine treatment plans. (6) The teacher analyzed and summarized the case.

The FSP and OSP-TCM group were exposed to the same cases used in the control group, and the training process for both groups consisted of six steps [17, 18]. (1) The teacher delivered lectures on the diseases involved in each case. (2) Students participated in open discussion. (3) The FSPs or OSPs introduced students to information on the patient’s personal data and disease conditions based on the clinical cases, and demonstrated TCM-specific symptoms and signs via images and medical devices. (4) The SP provided feedback and advice to the student on their consultation and physical examination. (5) Students completed a medical history, (the results of TCM syndrome differentiation and disease), and established a Chinese medicine treatment plan (treatment methods, rules, prescriptions, dosage, and medicine usage). (6) The teacher analyzed and summarized the case. It is worth noting that OSPs is derived from our previous study.

The study started in February 2023 and ended in October 2023. Volunteer recruitment and training were completed prior to the start of the course. The course started in March 2023 and evaluated and examined students at the end of the course. The data was collected from those who did not participate in this study and analyzed by the professionals.

### Evaluation of training effectiveness

#### Formative evaluation

The formative evaluation utilized a refined adaptation of the mini-Clinical Evaluation Exercise (mini-CEX) used in our previous study [22], with the assessments conducted on a nine-point scale (Supplement 3). This evaluation encompassed five domains employed to assess the clinical competence of the participants: physical examination, medical interview, disease treatment, clinical judgment, and overall performance (eTable 1 in Supplement 4). This session took place in the middle of the curriculum (6th week).

#### Summative evaluation

##### Online systematic test

The assessment format for this stage was an online case exam comprising six cases (100-point scale). The first five cases were multiple-choice questions, each with five questions covering disease diagnosis, syndrome differentiation, treatment principles, treatment methods, and major prescriptions. The final case required students to analyze the given case and provide answers to questions on TCM diagnosis, syndrome differentiation, the basis of syndrome differentiation, treatment principles, prescriptions, and analysis of the chosen prescription. The entire exam lasted for 90 min.

##### Offline clinical skill test

OSPs to obtain information on their medical history. Subsequently, within a 30-minute timeframe, they completed treatment based on syndrome differentiation and medical records. The results of interviews, medical records, and differential diagnosis treatment obtained during this process were evaluated by six TCM professionals not involved in teaching based on pre-established criteria. Evaluation criteria, including the application of TCM skills, written medical records, TCM syndrome differentiation, and therapeutic regimen are provided in eTable 2 in Supplement 4.

#### Real time assessment

The Arizona Clinical Interview Rating (ACIR) scale was used to evaluate the communication ability and interviewing skills of the students [17]. This standardized assessment comprised 20 items, each assigned a numerical value ranging from 1 to 5, where scores of 1 and 5 represented poor and excellent performances, respectively. Once students collected the medical data, OSPs evaluated and assigned scores for each item.

#### Feedback questionnaire

Three different questionnaire feedback forms were designed to capture the perceptions and opinions of students, SPs, and teachers. After course completion, we administered a questionnaire to the three groups of students. The questionnaire aimed to gather feedback on their attitudes towards the course and identify any benefits they had accrued for optimization of future teaching initiatives. Furthermore, we surveyed 12 participating teachers to ascertain their views and suggestions on the use of FSP-TCM in clinical skills training. Concurrently, we administered a questionnaire to the 20 SP volunteers involved in this study to gain insight into their impressions of the study and their willingness to continue playing the role of an SP. By exploring each participant group’s insights and perspectives, we sought to promote ongoing improvement in both SP training and its practical application.

#### The cost comparison between OSP-TCM and FSP-TCM

To enable a comparison of cost between the FSP-TCM and OSP-TCM training modes, detailed expenditure records were meticulously documented. These records covered expenses encompassing training expense, qualification authentication, course fees, transportation allowances, retraining expense, re-qualification authentication, medical examinations, and psychological assessments.

### Statistical analysis

SPSS 27.0 software were used for processing and analysis the statistical data. In this study, continuous variables were presented as mean ± standard deviation. The normality of the data in each outcome indicator across the three groups was assessed using the Shapiro-Wilk test. In cases where the data deviated from a normal distribution, a rank sum test was employed for statistical analysis. Furthermore, when the data followed a normal distribution, a variance chi-square test was initially conducted prior to performing a one-way ANOVA. Subsequently, if the data met the assumption of variance chi-square, the Tukey test was utilized for further comparative analyses. Conversely, if the assumption of variance chi-square was not met, the Dunnett’ 3 method was employed. Dichotomous variables were analyzed using frequencies and percentages, and statistical significance was assessed using the chi-square test (*P* < 0.05 was considered statistically significant).

## Results

### The basic characteristics of participants

Ninety students participated in this study, and they were randomly divided into three groups: FSP-TCM (*n* = 30), OSP-TCM (*n* = 30), and TT (*n* = 30) in a 1:1:1 ratio, following the principle of randomization. No statistically significant differences among the members of the three groups in terms of sex (*P* = 0.72), age (*P* = 0.96), basic Chinese medicine courses, and basic Western medicine courses were found. The detailed basic information of the participating students is shown in Table 1.

**Table 1:** The baseline characteristics of the participants (*N* = 90) ;

Demographics	Traditionally taught group	FSP-TCM group	OSP-TCM group	*F* Value/χ2	*P* Value
	(*n* = 30)	(*n* = 30)	(*n* = 30)		
Age (year), mean ± SD	21.37 ± 0.77	21.27 ± 0.94	21.17 ± 1.12	0.33	0.72
**Gender**, ***n*(%)**					
Female	16(53.33%)	16 (53.33%)	15 (50%)	0.089	0.96
Male	14(46.67%)	14 (46.67%)	15 (50%)		
**Basic courses of Traditional Chinese Medicine**, **mean ± SD**					
Fundamental theory of TCM	74.4 ± 4.15	73.63 ± 3.59	73.37 ± 4.35	0.53	0.59
Chinese materia medica	75 ± 3.60	75.2 ± 4.61	75.03 ± 4.32	0.02	0.98
Diagnostics of TCM	76.63 ± 2.14	76.8 ± 1.54	76.27 ± 1.80	0.66	0.52
Formulaology of TCM	76.2 ± 2.72	76.7 ± 2.10	75.77 ± 2.18	1.19	0.31
**Basic courses of Western Medicine**, **mean ± SD**					
Anatomy	75.8 ± 2.34	75.3 ± 2.25	75.03 ± 2.59	0.79	0.46
Physiology	74.4 ± 2.79	74.37 ± 2.90	74.47 ± 2.62	0.01	0.99
Pathology	74.3 ± 2.55	74.6 ± 2.62	74.47 ± 2.85	0.11	0.90
Medical biology	73.97 ± 2.85	75.07 ± 2.77	74.5 ± 2.84	1.14	0.32
Diagnostics of Western medicine	72.97 ± 3.91	72.27 ± 2.12	71.53 ± 2.11	1.91	0.15

Abbreviations: TCM, Traditional Chinese Medicine; FSP-TCM, faculty standardized patients of traditional Chinese medicine; OSP-TCM, occupational standardized patients of traditional Chinese medicine

### Evaluation of training effectiveness

#### Formative evaluation

**Fig. 1:**
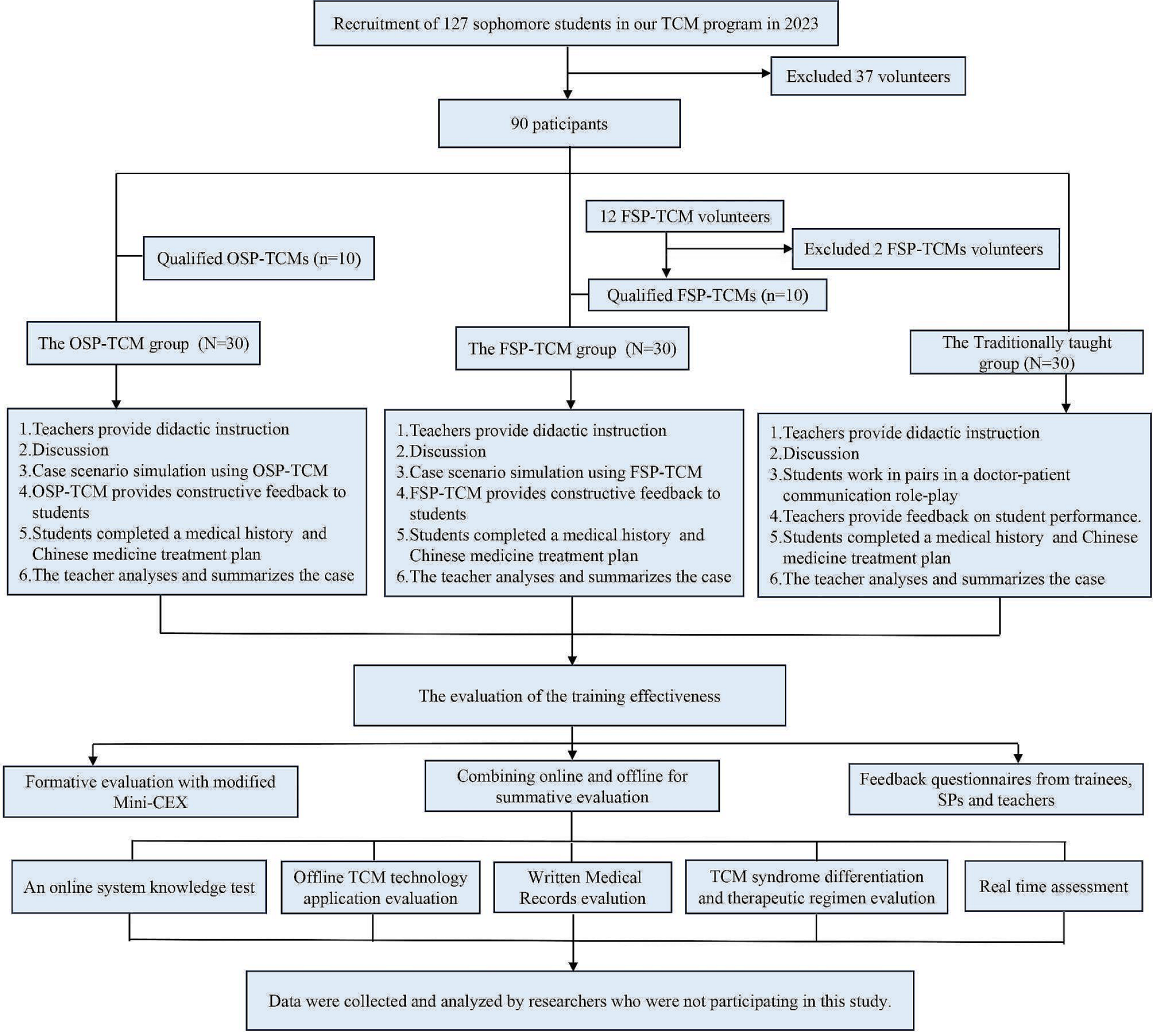
Flowchart of this study

The results shown in Fig. 1 indicate a consistent trend across all five dimensions (medical interview, physical examination, clinical judgment, disease treatment, and comprehensive), with slightly higher scores observed in the SP-TCM groups compared to the TT group. Specifically, focusing on medical interviews revealed that data from all three groups followed a normal distribution and showed homogeneity of variance (*P* = 0.84). Furthermore, no significant statistical differences among the three groups were found (F = 4.14, *P* = 0.02).

The OSP-TCM group demonstrated superior performance compared to both FSP-TCM and TT groups in terms of medical interview scores, with significant difference between OSP-TCM and TT groups (TT: mean 6.43 SD 0.94, FSP-TCM: 7.03 SD 0.93, OSP-TCM: mean 7.07 SD 0.98, *P*^a^ = 0.04, *P*^b^ = 0.03). However, no statistically significant difference observed between FSP-TCM and OSP-TCM groups (*P*^c^ = 0.99). Moreover, in terms of physical examination, the observed variation between TT and FSP-TCM and OSP-TCM groups had statistically significant (*P*^a^ = 0.01; *P*^b^ = 0.04, respectively) (TT: mean 5.73 SD 1.11, FSP-TCM: mean 6.53 SD 0.90, OSP-TCM: mean 6.43 SD 1.19, *P*^c^ = 0.93).

**Fig. 2:**
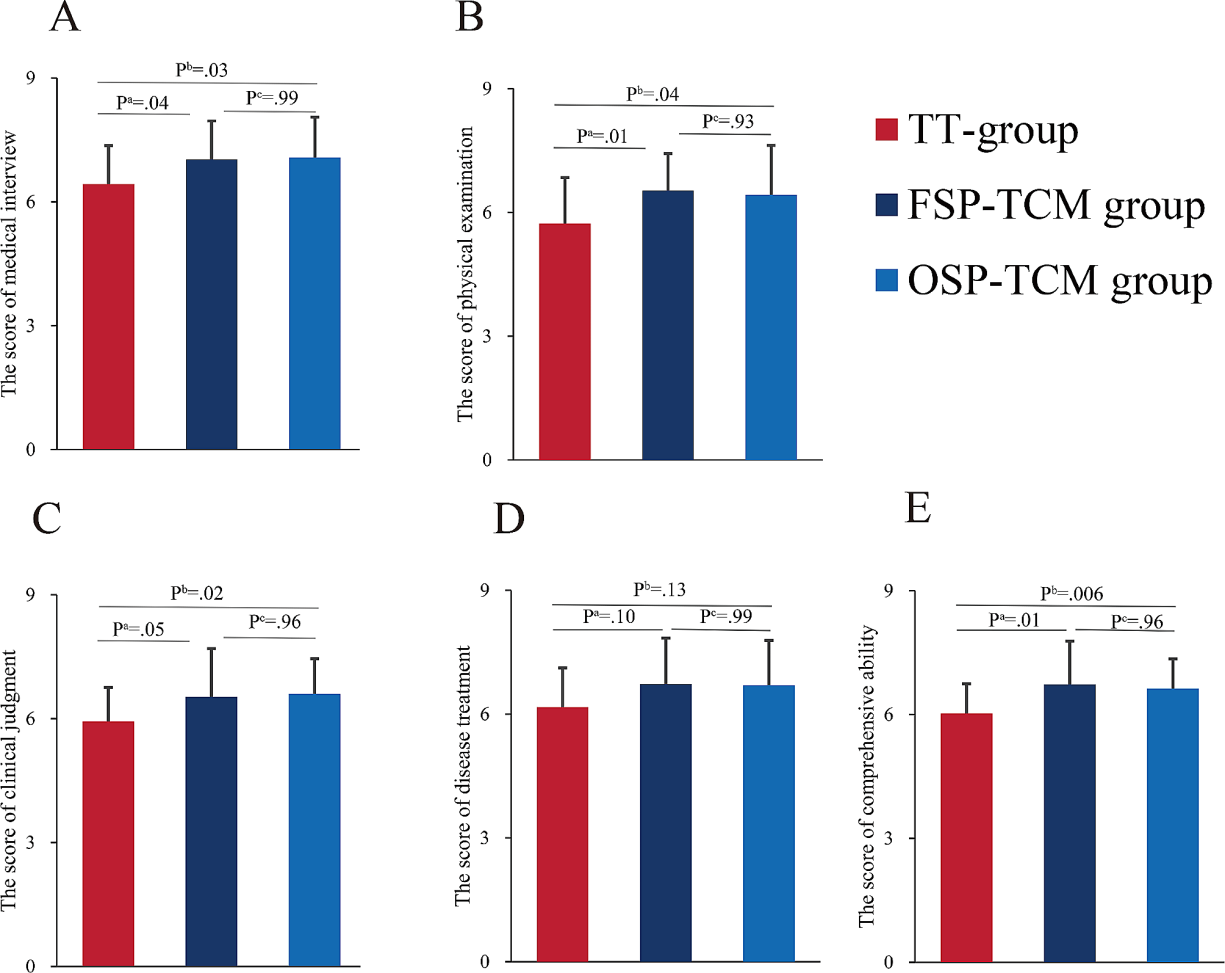
The score of formative evaluation. (**A**) Score of medical interview. (**B**) Score of physical examination. (**C**) Score of clinical judgment. (**D**) Disease treatment. (**E**) Score of comprehensive ability Notes: ^a^ indicates a significant difference between FSP-TCM and TT group (*P* < 0.05) ^b^ indicates a significant difference between OSP-TCM and TT group. (*P* < 0.05)

The same trend was found for comprehensive ability and clinical judgment (Comprehensive ability: TT: mean 6.03 SD 0.718, FSP-TCM: 6.73 SD 1.05, OSP-TCM: mean 6.63 SD 0.72, *P*^a^ = 0.01, *P*^b^ = 0.006, *P*^c^ = 0.96; clinical judgment: TT: mean 5.93 SD 0.83, FSP-TCM: mean 6.53 SD 1.17, OSP-TCM: mean 6.6 SD 0.86, *P*^a^ = 0.05, *P*^b^ = 0.02, *P*^c^ = 0.96). Both SP groups outperformed the TT group in disease treatment scores, and no statistically significant distinction among the three groups (TT: mean 6.17 SD 0.95, FSP-TCM: mean 6.73 SD 1.11, OSP-TCM: mean 6.7 SD 1.09, *P*^a^ = 0.10, *P*^b^ = 0.13, *P*^c^ = 0.99) was found (Fig. 2).

#### Summative evaluation

##### Online systematic knowledge test

**Fig. 3:**
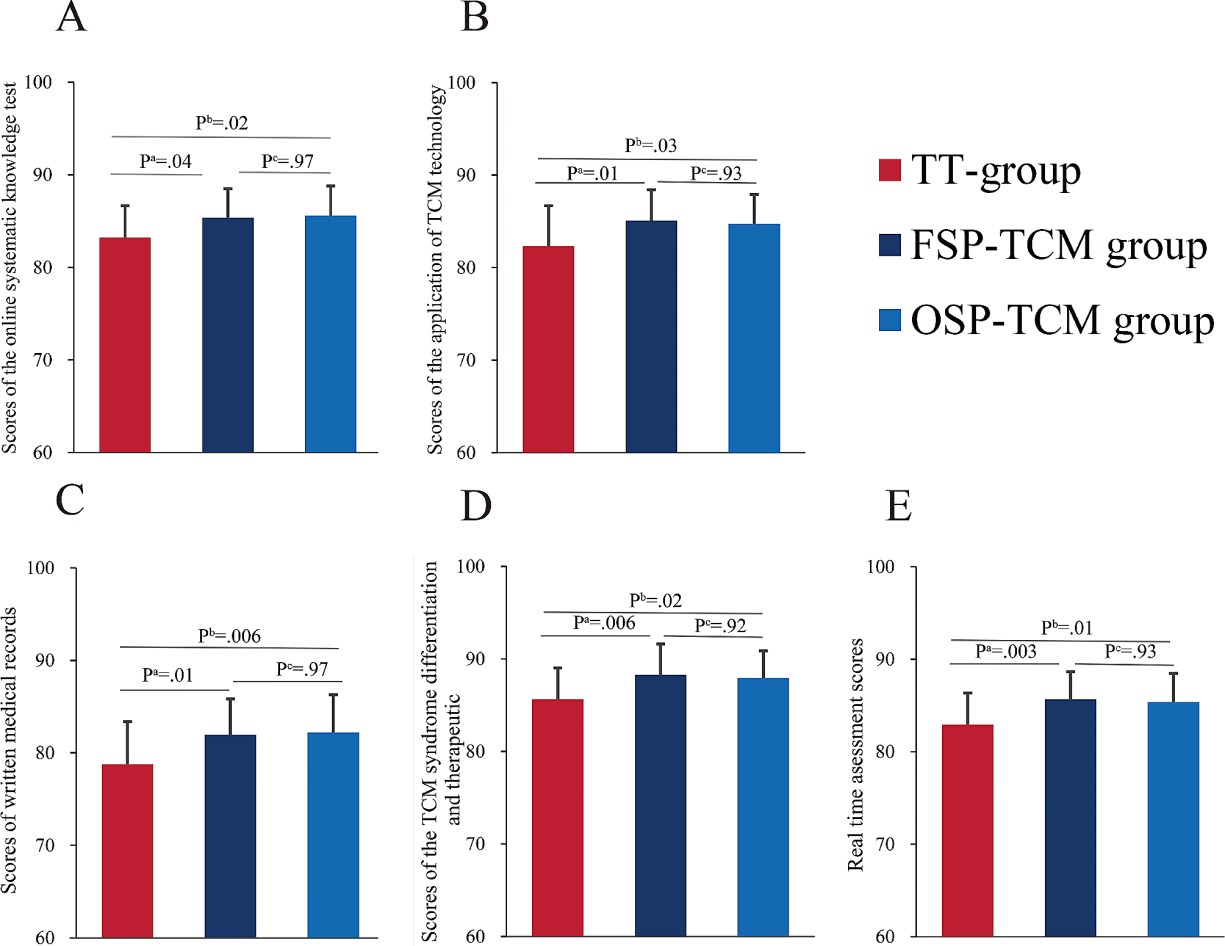
The score of summative evaluation. (**A**) Score of online systematic knowledge test. (**B**) Score of the application of TCM technology. (**C**) Score of written medical records. (**D**) Score of TCM syndrome differentiation and therapeutic. (**E**) Real-time assessment scores Notes: ^a^ indicates a significant difference between FSP-TCM and TT group (*P* < 0.05) ^b^ indicates a significant difference between OSP-TCM and TT group. (*P* < 0.05)

Based on the results of the Shapiro-Wilk test, the online systematic knowledge test data of all three groups conformed to a normal distribution. The chi-square test showed that the variances of the three groups were equal (*P* = 0.94). The two SP groups scored greater than the control group. The results of one-way ANOVA showed that the online systematic knowledge test data of the three groups were different, with statistically significant differences (F = 4.71, *P* = 0.01). The FSP-TCM and OSP-TCM groups outperformed the TT group in terms of online systematic knowledge test scores (TT: mean 83.23, SD 3.43; FSP-TCM: mean 85.37, SD 3.14, OSP-TCM: mean 85.57, SD 3.22, *P*^a^ = 0.04, *P*^b^ = 0.02). However, no statistically significant differences between the FSP-TCM and OSP-TCM groups were found (*P*^c^ = 0.97) as shown in Fig. 2 (Note: *P*^a^ = FSP-TCM group vs. TT group, *P*^b^=OSP-TCM group vs. TT group, *P*^c^=FSP-TCM group vs. OSP-TCM group) (Fig. 3).

##### Application of TCM technology

Shapiro-Wilk test indicated that the scores of the application of TCM technology data within all three groups adhered to a normal distribution. Moreover, the results of the chi-square test showed that the variances of the three groups were equal (*P* = 0.21). Results of one-way ANOVA showed that the scores of the application of TCM technology data of the three groups were significantly different (F = 4.97, *P* = 0.009). The scores of FSP-TCM group were better than those of the TT group (TT: mean 82.33, SD 4.37; FSP-TCM: mean 85.07, SD 3.33, *P*^a^ = 0.01), and those of OSP-TCM group were higher than the TT group (OSP-TCM: mean 84.73, SD 3.18, *P*^b^ = 0.03), with a statistically significant difference. However, there was no significant difference between the OSP-TCM and FSP-TCM groups (*P*^c^ = 0.93).

##### Scores of written medical records

Shapiro-Wilk test indicated that the scores of written medical records data of all three groups adhered to a normal distribution. The chi-square test showed that the variances of the three groups were equal (*P* = 0.29). One-way ANOVA showed that the scores of written medical records data of the three groups were significantly different (F = 6.17, *P* = 0.003). The scores of the FSP-TCM and OSP-TCM groups were better than that of the TT group (TT: mean 78.77 SD 4.61; FSP-TCM: mean 81.93 SD 3.90, OSP-TCM: mean 82.20 SD 4.09, *P*^a^ = 0.01, *P*^b^ = 0.006, *P*^c^ = 0.97) but no significant difference between the FSP-TCM and TT groups was found. Furthermore, the difference between the FSP-TCM and OSP-TCM groups was not statistically significant.

##### Scores of the TCM syndrome differentiation and therapeutic regimen

Shapiro-Wilk test indicated that the scores of the TCM syndrome differentiation and therapeutic regimen of all three groups adhered to a normal distribution. The variances of the three groups were equal (*P* = 0.74). One-way ANOVA showed that the differences among the three groups were statistically significant (F = 5.944, *P* = 0.004). The scores of the FSP-TCM and OSP-TCM groups were higher than that of the TT group (TT: mean 85.63 SD 3.39; FSP-TCM: mean 88.27 SD 3.33; OSP-TCM: mean 87.93 SD 2.924; *P*^a^ = 0.006, *P*^b^ = 0.02). However, the same trend was found between the FSP-TCM and OSP-TCM groups, with no significant difference (*P*^c^ = 0.92).

##### Real time assessment scores

The Shapiro-Wilk test indicated that the real time assessment scores data of all three groups adhered to a normal distribution. The variances of the three groups were equal (*P* = 0.77). One-way ANOVA showed that the differences among the three groups were statistically significant (F = 6.73, *P* = 0.002). The same trend was observed among the three groups. The scores of the FSP-TCM and OSP-TCM groups were higher than the TT group (TT: mean 82.93 SD 3.41, FSP-TCM: mean 85.67 SD 2.96, OSP-TCM: mean 85.37 SD 3.09; *P*^a^ = 0.003, *P*^b^ = 0.01, *P*^c^ = 0.93).

#### Feedback questionnaire

##### Student feedback questionnaire analysis

Table 2 summarizes the results of the student questionnaire feedback. The students in the FSP and SP-TCM groups showed higher levels of approval for this course compared to those in the TT group in the “satisfaction with the course”, “confidence in handling clinical work,” (χ²=11.09, *P*^a^ = 0.03; χ²=10.54, *P*^b^ = 0.03) and “Motivation to study TCM” (χ²=10.06, *P*^a^ = 0.04; χ²= 11.399, *P*^b^ = 0.02). Moreover, trainees in the two SP-TCM groups made greater progress in medical information processing relative to the TT group, in terms of “knowledge of medical history,” (χ²=10.83, *P*^a^ = 0.03; χ²=11.247, *P*^b^ = 0.02) “ability to write medical records,”(χ²=15.13, *P*^a^ = 0.004, χ²=13.07, *P*^b^=.01) and “ability to syndrome differentiation and treatment” (χ²=9.51, *P*^a^=.05; χ²=12.70, *P*^b^=.01).

**Table 2:** Results of student feedback questionnaire analysis

Items	FSP-TCM group (*n*, %)					OSP-TCM group (*n*, %)					Traditionally taught group (*n*, %)					*P*-value(X ²)a	*P*-value(X ²)b	*P*-value(X ²)c
	Strong agree	agree	Neutral	Disagree	Strong disagree	Strong agree	Agree	Neutral	Disagree	Strong disagree	Strong agree	Agree	Neutral	Disagree	Strong disagree			
You are highly satisfied with the course.	10(33.33)	15(50.00)	2(6.67)	2(6.67)	1(3.33)	10(33.33)	16(53.33)	2(6.67)	1(3.33)	1(3.33)	4(13.33)	9(30.00)	6(20.00)	7(23.33)	4(13.33)	0.03(10.65)	0.02(12.98)	0.99(0.37)
The course increased your confidence in handling clinical work.	11(36.67)	14(46.67)	3(10.00)	2(6.67)	0(0.00)	12(40.00)	13(43.33)	2(6.67)	2(6.67)	1(3.33)	6(20.00)	7(23.33)	9(30.00)	5(16.67)	3(10.00)	0.03(11.09)	0.03(10.54)	0.87(1.28)
The course increased your motivation in learning TCM.	7(23.33)	16(53.33)	4(13.33)	2(6.67)	1(3.33)	9(30.00)	14(46.67)	14(46.67)	2(6.67)	0(0.00)	4(13.33)	7(23.33)	9(30.00)	6(20.00)	4(13.33)	0.04(10.06)	0.02(11.40)	0.83(1.49)
The course enhanced your integrity of medical history acquisition.	9(30.00)	13(43.33)	5(16.67)	3(10.00)	0(0.00)	11(36.67)	12(40.00)	4(13.33)	2(6.67)	1(3.33)	3(10.00)	8(26.67)	9(30.00)	5(16.67)	5(16.67)	0.03(10.83)	0.02(11.25)	0.82(1.55)
The course enhanced your TCM clinical skills.	12(40.00)	14(46.67)	2(6.67)	1(3.33)	1(3.33)	13(43.33)	12(40.00)	3(10.00)	2(6.67)	0(0.00)	5(16.67)	9(30.00)	11(36.67)	3(10.00)	2(6.67)	0.02(11.53)	0.03(10.76)	0.35(4.46)
The course enhanced your interpersonal communication skills.	11(36.67)	15(50.00)	2(6.67)	1(3.33)	1(3.33)	10(33.33)	13(43.33)	4(13.33)	2(6.67)	1(3.33)	4(13.33)	7(23.33)	8(26.67)	7(23.33)	4(13.33)	0.003(16.08)	0.03(10.28)	0.88(1.12)
The course cultivated your ability in TCM syndrome differentiation and treatment.	9(30.00)	14(46.67)	4(13.33)	2(6.67)	1(3.33)	7(23.33)	11(36.67)	6(20.00)	4(13.33)	2(6.67)	4(13.33)	8(26.67)	10(33.33)	5(16.67)	3(10.00)	0.05(9.51)	0.01(12.70)	0.96(0.67)
The course enhanced your ability to write medical records.	12(40.00)	15(50.00)	2(6.67)	1(3.33)	0(0.00)	10(33.33)	16(53.33)	2(6.67)	2(6.67)	0(0.00)	7(23.33)	9(30.00)	7(23.33)	5(16.67)	2(6.67)	0.004(15.13)	0.01(13.07)	0.91(0.55)
The course enhanced your ability to build harmonious doctor-patient relationships.	9(30.00)	12(40.00)	5(16.67)	3(10.00)	1(3.33)	8(26.67)	14(46.67)	5(16.67)	2(6.67)	1(3.33)	3(10.00)	8(26.67)	9(30.00)	7(23.33)	3(10.00)	0.005(14.78)	0.02(11.79)	0.93(0.88)

Abbreviation: Traditional Chinese Medicine; TT, Traditionally taught; *P*^a^ = FSP-TCM group vs. TT group, *P*^b^ = OSP-TCM group vs. TT group, *P*^c^ = FSP-TCM group vs. OSP-TCM group

Students in the FSP and OSP-TCM groups were more positive than those in the TT group regarding the enhancement of doctor-patient communication skills, “interpersonal skills,” (χ²=16.08, *P*^a^ = 0.003; χ²=10.28, *P*^b^ = 0.03) and “ability to build harmonious doctor-patient relationships” (χ²=14.78, *P*^a^=.005; χ²=11.79, *P*^b^=.02). Similar trends were observed for the clinical skills required for Chinese medicine (χ²=11.533, *P*^a^=.02; χ²=10.76, *P*^b^=.03). However, no significant differences in feedback between students in the FSP and OSP-TCM groups across these 11 items were found.

##### Feedback questionnaire analysis of SPs

Eight items were set to obtain information about the feelings and self-evaluations of the SP volunteers who participated during the course (Table 3). Volunteers of both groups showed a strong willingness to “continue the training course as SP-TCM” (FSPs: strongly agree 40%, agree 50% vs. OSPs: strongly agree 60%, agree 40%, χ²=1.51, *P* = 0.47). For switching roles, only a small number of FSPs and OSPs thought that there was difficulty (FSPs: agree 10% vs. OSPs: agree 10%, χ²=2.25, *P* = 0.52), and for “providing a flexible clinical environment,” more than half of the volunteers were in favor (FSPs: strongly agree 20%, agree 50% vs. OSPs: strongly agree 30%, agree 40%, χ²=1.64, *P* = 0.80).

**Table 3:** Results of SPs feedback questionnaire

Item	FSPs group (*n*, %)					OSPs group (*n*, %)					
	strong agree	agree	neutral	disagree	strong disagree	strong agree	agree	neutral	disagree	strong disagree	*P*-value(X²)
Are you willing to serve as SP-TCM to engage in the simulation training course?	4(40)	5(50)	1(10)	0(0)	0(0)	6(60)	4(40)	0(0)	0(0)	0(0)	0.47(1.51)
Was it difficult to transform your role to SP-TCM?	0(0)	1(10)	2(20)	4(40)	3(30)	0(0)	1(10)	0(0)	5(50)	4(40)	0.52(2.25)
Did you provide live and flexible clinical settings?	2(20)	5(50)	1(10)	2(20)	0(0)	3(30)	4(40)	1(10)	1(10)	1(10)	0.80(1.64)
Did our performance achieve a high-fidelity rate as a “real patient”?	1(10)	3(30)	3(30)	2(20)	1(10)	2(20)	4(40)	2(20)	1(10)	1(10)	0.91(1.01)
Did you precisely present typical symptoms and signs of TCM syndrome?	1(10)	3(30)	3(30)	2(20)	1(10)	2(20)	5(50)	1(10)	2(20)	0(0)	0.59(2.83)
Did you give some hints to the students in the simulation training?	1(10)	3(30)	3(30)	2(20)	1(10)	0(0)	1(10)	2(20)	4(40)	3(30)	0.42(3.87)
Was the constructive feedback you provided professional?	3(30)	5(50)	1(10)	1(10)	0(0)	2(20)	4(40)	1(10)	3(30)	1(10)	0.69(2.27)
Did you use any medical jargons in the simulation training	1(10)	3(30)	2(20)	2(20)	2(20)	0(0)	1(10)	1(10)	4(40)	4(40)	0.45(3.67)

Abbreviations: TCM, Traditional Chinese Medicine; FSPs, faculty standardized patients; OSPs, occupational standardized patients

When evaluating the fidelity of their own performances as SPs compared to real patients, OSPs showed greater recognition compared to FSPs (FSPs: strongly agree 10%, agree 30% vs. OSPs: strongly agree 20%, agree 40%, χ²=1.01, *P* = 0.91). There was a comparable trend in “presentation of Chinese medicine syndrome” (FSPs: strongly agree 10%, agree 30% vs. OSPs: strongly agree 20%, agree 50%, χ²=2.83, *P* = 0.59). It was noteworthy that more FSPs than OSPs thought they provided “professional and constructive feedback” during the course (FSPs: strongly agree 30%, agree 50% vs. OSPs: strongly agree 20%, agree 40%, χ²=2.27, *P* = 0.69). In contrast, more FSPs than OSPs indicated that they gave hints and used medical terminology for students during the course (FSPs: strongly agree 10%, agree 30% vs. OSPs: strongly agree 0%, agree 10%, χ² = 3.867 *P* = 0.42; χ² = 3.67, *P* = 0.45).

##### Analysis of the teacher feedback questionnaire

Analysis of the teacher feedback questionnaire is presented in Table 4. Overall, teachers who participated in the questionnaire feedback favored FSPs in this course. Ten (83%) teachers expressed willingness to continue using FSP-TCM simulation for clinical training, and 11 out of 12 teachers (91.66%) agreed that the use of FSP-TCM could effectively supplement bedside teaching and reduce teaching costs.

**Table 4:** Results of teacher feedback questionnaire

Item	(*n*, %)				
	Strong agree	Agree	Neutral	Disagree	Strong disagree
Are you willing to continue to use FSP-TCM simulation for clinical training?	3(25.00)	7(58.33)	1(8.33)	1(8.33)	0(0.00)
Do you agree that FSP-TCM simulation is a good supplement to bedside teaching?	3(25.00)	8(66.67)	0(0.00)	1(8.33)	0(0.00)
Do you agree that FSP-TCM simulation can reduce teaching costs of clinical training?	4(33.33)	7(58.33)	1(8.33)	0(0.00)	0(0.00)
Do you agree that FSP-TCM simulation can improve teaching efficiency of clinical training?	1(8.33)	10(83.3)	0(0.00)	1(8.33)	0(0.00)
Do you agree that FSP-TCM simulation can enhance students’ syndrome differentiation and treatment ability?	7(58.33)	4(33.33)	1(8.33)	0(0.00)	0(0.00)
Do you agree that FSP-TCM simulation can enhance students’ motivation in learning TCM?	8(66.67)	3(25.00)	1(16.67)	0(0.00)	0(0.00)
Do you agree that FSP-TCM simulation can enhance students’ critical thinking on TCM?	3(25.00)	6(50.00)	2(16.67)	1(8.33)	0(0.00)
Do you agree that case script of FSP-TCM simulation should be constructed primarily based on different TCM syndromes?	4(33.33)	6(50.00)	2(16.67)	0(0.00)	0(0.00)
Do you agree that case script of FSP-TCM simulation should be constructed primarily based on different diseases?	1(8.33)	2(16.67)	2(16.67)	4(33.33)	3(25.00)

Abbreviations: TCM, Traditional Chinese Medicine; FSP-TCM, faculty standardized patients of Traditional Chinese Medicine

Regarding teaching effectiveness, FSP-TCM simulation improved teaching efficiency of clinical training (strongly agree: 1, 8.33%; agree: 10, 83.3%), enhanced students’ syndrome differentiation and treatment ability (strongly agree: 7, 58.33%; agree, 4 33.33%), improved students’ critical thinking on TCM (strongly agree: 3, 25.0%; agree: 6, 50.0%), and motivation to learn TCM (strongly agree: 8, 66.67%; agree: 3, 25.0%). Regarding the construction of case scripts for FSP-TCM simulation, teachers preferred to various TCM syndromes (strongly agree: 4, 33.33%; agree: 6, 50.0%) rather than different diseases (strongly agree: 1, 8.33%; agree: 2, 16.67%).

#### Analysis of cost comparison between FSP-TCM and OSP-TCM

The expenses for qualification authentication (one time /per person, $27.60), re-qualification authentication (one time biennially/per person, $27.60), and psychological assessment (one time biennially/per person, $41.40) are identical for both FSPs and OSPs. However, the two models diverge in terms of five key expense categories: training expenses, course fees, traffic allowance, retraining expenses, and medical examinations. Notably, the training and retraining expenses for OSPs are double those of FSPs (training expense: FSPs $345.00 vs. OSPs $690.00; retraining expense: FSPs $69.00 vs. OSPs $138.00). As for course fees, FSPs incur a cost of $6.90 per person per session, significantly lower than the $16.56 per person per session cost for OSPs. This disparity arises from FSPs’ participation being considered a teaching assignment, leading to a reduced classroom fee in comparison to OSPs. Furthermore, as faculty members undergo annual school-organized medical examinations, the cost of medical examinations for FSPs is excluded, with OSPs incurring a fee of $41.40 per person biennially. Additionally, each OSP receives a $6.90 transportation allowance per course attended.

In conclusion, the total cost for FSP-TCM amounts to $7590.00, significantly lower than the total cost of $17415.60 for OSP-TCM, highlighting the cost-effectiveness of the FSP-TCM simulation training model in TCM education (supplement 5).

## Discussion

Formative assessment revealed a significant improvement in the overall competence of trainers with the FSP and OSP-TCM groups compared to the TT group, particularly in medical interviews and physical examinations. While no significant differences were noted in clinical judgment and disease treatment, the benefits of employing FSP and OSP training methods became more apparent during summative assessment. Students using FSPs and OSPs showed significant improvements in their knowledge of the system, ability to write medical records, ability to apply Chinese medicine techniques, and accuracy of their diagnoses and treatment.

Furthermore, over 80% of the participants in both the FSP and OSP-TCM groups acknowledged that this course significantly enhanced their proficiency in TCM clinical skills. Unlike traditionally taught methods, SP-TCM simulation utilizes SP as a bridge to construct a “simulated clinical environment” for trainees based on specific case scripts, vividly presenting other monotonous disease characteristics. The SP-TCM method enhances disease concreteness and characteristics, deepens students’ understanding of diseases and medical history, and improves their ability to differentiate and treat syndromes and proficiency in TCM [23, 24]. By using SPs instead of real patients, the trainees can obtain diagnostically beneficial information through methods, including “observation, listening, inquiry, and pulse examination,” nurturing their communication and interpersonal skills and preparing them for establishing harmonious doctor-patient relationships in the future [25]. The training mode using SPs as a substitute for real patients provides a safe environment for students [26], thereby effectively preventing the risks that students may encounter in a real medical setting [27].

The advantages of FSPs are not limited to these aspects. FSPs are teachers or doctors who have been teaching for more than 5 years, so compared to OSPs who do not have a medical background, it is easier for them to understand the medical terminology of Chinese medicine, thus greatly reducing the duration of the training cycle (FSPs: 20 of credit hours vs. OSP: 40 of credit hours). Furthermore, FSPs are more professional in giving students constructive feedback during this study compared to OSPs (FSPs: 30% Strongly agree, 50% agree vs. OSPs: 20% strongly agree, 40% agree). Meanwhile, as faculty members, they have lesser job mobility and can continue to participate in simulation training as SPs stably [28]. OSP training incurs high costs, including appearance fees, transportation allowances, and social insurance [29]. Conversely, FSPs effectively mitigate these cost-related challenges. The overall expense analysis revealed a significant cost advantage during the research process between FSP-TCM (¥55000.00/$7590.00) and OSP-TCM (¥126200.00/$17415.60). Utilizing FSP-TCM can result in a savings of $9825.6 compared to the OSP-TCM training mode, illustrating significant cost-effectiveness. This advantage primarily stems from differences in training and retraining durations, medical examination, course fees, and transportation allowances among the two groups of volunteers.

However, the results from the SP feedback questionnaire reflected some issues regarding FSPs. Specifically, 40% (10% strongly agree, 30% agree) of these FSPs felt that they unconsciously gave students hints and used medical terminology during simulation training, and 10% of OSPs indicated a similar situation. Some factors may contribute to this phenomenon.

FSPs, as medical faculty, already possess extensive medical knowledge and inevitably encounter a vast array of medical terminology in their daily work. This immersion in the field and habitual influence leads to the unconscious integration of medical terms into their daily communication. Additionally, they assume the role of teachers during their regular teaching activities [30]. When interacting with students, they may instinctively employ cues to steer them toward the correct answers [31]. Other studies reported the similar shortcomings of OSPs and SSPs [21, 32]. It is noteworthy is that this phenomenon diminished with increased training experience. In the subsequent phase, enhancing FSPs’ understanding of patient roles through targeted training and coaching should be prioritized to diminish the use of medical terminology and jargon.

### Limitations

This study recruited teaching staff as FSPs for participation in simulated training of students’ clinical abilities, achieving positive results. However, some limitations of the study warrant consideration. First, FSPs taking on multiple roles during training may encounter difficulties in role switching, resulting in problems for students in implication and use of medical terminology. However, these problems can be overcome through long-term targeted training and guidance. Second, this study is prospective and a follow-up on the clinical abilities of the trainees was lacking, thus providing limited knowledge about the post-training capabilities of the participants. Third, clinical patients exhibit diversity, including in terms of age groups, characteristics of different diseases, and TCM syndromes, which FSPs as teaching staff cannot fully represent.

## Conclusion

FSP-TCM training mode showed greater effectiveness than traditional teaching method in improving clinical competence among TCM students. It possesses certain characteristics that render it feasible, practical, and cost-effective, and thus, it presents a viable alternative to OSP-TCM simulation. Further optimization of FSP-TCM to facilitate its promotion and development is necessitated.

### Electronic supplementary material

Below is the link to the electronic supplementary material.

Supplement 1: FSP Recruitment Information

Supplement 2: Training Method of FSP-TCM and OSP-TCM

Supplement 3: Course arrangement. The Formative Evaluation Methods

Supplement 4: eTable 1: Modified Mini-CEX. eTable 2: The Scoring Details of the Offline Clinical Skill Test

Supplement 5: The Cost Comparison Between FSP-TCM and OSP-TCM

## Data Availability

The data generated or analyzed during this study are available in this published article and its supplementary information files.

## Declarations

### Clinical trial registration

The authors would like to clarify that this study does not fall under the category of clinical intervention study, as confirmed by consultation with the China Clinical Trial Registry (https://www.chictr.org.cn/). Our study, categorized as educational research, does not involve human trials or pharmaceuticals, thus exempting it from requiring a clinical registration ID.

## Abbreviations

ACIR: Arizona Clinical Interview Rating
CDUTCM: Chengdu University of TCM
FSP-TCM: Faculty SP for Traditional Chinese medicine
Mini-CEX: Mini-Clinical Evaluation Exercise
OSP-TCM: Occupational SP for Traditional Chinese Medicine
SPs: Standardized Patients
TCM: Traditional Chinese Medicine
TT Group: Traditionally Taught Group
