# Supplementary material for: Faculty standardized patients versus traditional teaching method to improve clinical competence among traditional Chinese medicine students: a prospective randomized controlled trial
Source: BMC Med Educ. 2024 Jul 24;24:793. doi: 10.1186/s12909-024-05779-3 (PMC11267817; doi:10.1186/s12909-024-05779-3)
Supplement: Supplementary file 1 — Supplement 1: FSP Recruitment Information [file 12909_2024_5779_MOESM1_ESM.docx]

**Supplement 1 FSP recruitment information**

The recruitment of FSP volunteers adhere to the following conditions:

1. Volunteers had to undergo physical and mental health assessments and be certified to be in good health.
2. Volunteers need to have been teaching in the field for 5 years or more.
3. Education: Master degree or above.
4. Age: Between 35 and 55 years old.
5. All volunteers had to be willing to sign both a confidentiality agreement and an informed consent form.
6. Volunteers needed to have enough available time to attend training sessions prior to the study’s commencement and throughout its duration.
