# Supplementary material for: Faculty standardized patients versus traditional teaching method to improve clinical competence among traditional Chinese medicine students: a prospective randomized controlled trial
Source: BMC Med Educ. 2024 Jul 24;24:793. doi: 10.1186/s12909-024-05779-3 (PMC11267817; doi:10.1186/s12909-024-05779-3)
Supplement: Supplementary file 2 — Supplement 2: Training Method of FSP-TCM and OSP-TCM [file 12909_2024_5779_MOESM2_ESM.docx]

**Supplement 2**

**Training method of OSP-TCM**

**The production of script:** First of all, it is necessary to select the case materials of common diseases in TCM internal medicine training, and the script writing work should be completed jointly by the instructors and OSP-TCMs. The script content included the simulation of the scenario, medical history, diagnosis of TCM and western medicine, examination, treatment, follow-up visit, improvement, etc. The OSPs language in the script must be concise and easy to understand, try to be colloquial, avoid the use of medical terms, and add appropriate expressions and action requirements. And should also cooperate with props and makeup techniques when necessary. The plot would be discussed by instructor’ team followed by a semi-structured interview with OSP-TCMs reported open-ended questions, and then the first draft was written by instructors.

**The training course catalogue:** The script was sent to the OSP-TCM volunteers to familiarize them with the plot for 7 days, during which the volunteers were organized to observe the typical cases for 2 times to feel the emotion, language and body language. A senior SP trainer adopted multimodality educational intervention consisted of didactic sessions and skills practice using standardized training materials (including clinical clerkships training sessions), which included approximately 10 class hours of theoretical teaching and 30 class hours of clinical field simulation training. During clerkship, volunteers received the Clinical Performance Examination (CPX) training, which utilized SPs who complained of symptoms and signs of diseases. CPX training allows for the examination of one case for 40 min. One SP trainer takes 10 min to complete the following process with a SP: inquiry the SP volunteer, and conduct the four aspects of TCM diagnosis and physical examinations as required. When the SP volunteers leave, the faculty explains the suspected disease and the diagnosis plan to the instructor, and then the instructor and SP trainer provide feedback. To minimize bias, all training sessions are conducted by the same instructor.

**Training method of FSP-TCM**

**The production of script:** First of all, it is necessary to select the case materials of common diseases in TCM internal medicine training, and the script writing work should be completed jointly by the instructors and FSP-TCMs. The script content included the simulation of the scenario, medical history, diagnosis of TCM and western medicine, examination, treatment, follow-up visit, improvement, etc. The FSPs language in the script must be concise and easy to understand, try to be colloquial, avoid the use of medical terms, and add appropriate expressions and action requirements. And should also cooperate with props and makeup techniques when necessary. The plot would be discussed by instructor’ team followed by a semi-structured interview with FSP-TCMs reported open-ended questions, and then the first draft was written by instructors.

**The training course catalogue:** The script was sent to the faculties to familiarize them with the plot for 7 days, during which the faculties were organized to observe the typical cases for 2 times to feel the emotion, language and body language. A senior SP trainer adopted multimodality educational intervention consisted of didactic sessions and skills practice using standardized training materials (including clinical clerkships training sessions), which included approximately 4 class hours of theoretical teaching and 16 class hours of clinical field simulation training. During clerkship, faculties received the Clinical Performance Examination (CPX) training, which utilized SPs who complained of symptoms and signs of diseases. CPX training allows for the examination of one case for 40 min. One SP trainer takes 10 min to complete the following process with a SP: inquiry the SP volunteer, and conduct the four aspects of TCM diagnosis and physical examinations as required. When the SP volunteers leave, the faculty explains the suspected disease and the diagnosis plan to the instructor, and then the instructor and SP trainer provide feedback. To minimize bias, all training sessions are conducted by the same instructor.

**The qualification of SP-TCM**

After completing all training courses, all SP-TCM volunteers underwent qualification testing administered by senior SP instructors (unaware of OSP-TCM or FSP-TCM) for assessment.
