# Supplementary material for: Faculty standardized patients versus traditional teaching method to improve clinical competence among traditional Chinese medicine students: a prospective randomized controlled trial
Source: BMC Med Educ. 2024 Jul 24;24:793. doi: 10.1186/s12909-024-05779-3 (PMC11267817; doi:10.1186/s12909-024-05779-3)
Supplement: Supplementary file 3 — Supplement 3: Course arrangement. The Formative Evaluation Methods [file 12909_2024_5779_MOESM3_ESM.docx]

**Supplement 3**

**Course arrangement**

| Week | Course |
| --- | --- |
| The first week | Cough |
| The second week | Palpitation |
| The third week | Headache |
| The fourth week | Stomach pains |
| The fifth week | Edema |
| The sixth week | Diabetes |
| The seventh week | Lung distention |
| The eighth week | Insomnia |
| The ninth week | Diarrhea |
| The tenth week | Jaundice |
| The eleventh week | Fatigue |
| The twelfth week | Tympanites |

**Formative evaluation flow.**

1. After the sixth course teaching, all the students were assigned to each clinical department of the Hospital of Chengdu University of Traditional Chinese Medicine for practice by using the random number method. The selected clinical departments include respiratory department (disease: cough), cardiology department (disease: palpitation), neurology department (disease: headache), digestive department (disease: stomach pain), nephrology department (disease: edema), and endocrinology department (disease: diabetes).
2. Considering that the evaluation results of Mini CEX scale are easily affected by the subjective factors of different evaluators, we conducted systematic training
3. For residents in the above departments in advance to minimize the subjective bias caused by the evaluation of different doctors. After the training, a resident who did not participate in the evaluation would receive a standardized patient, and the trained resident will score the diagnosis and treatment according to the modified Mini-CEX. We calculated intragroup correlation coefficients (ICC) to evaluate the consistency among residents. When ICC>0.75, we thought the reliability among residents was high, and agreed they to participate in the following evaluation.
4. Each student carried out systematic diagnosis and treatment activities for patients, including medical history collection, physical examination, clinical diagnosis and development of diagnosis and treatment plan (including auxiliary examination, western medicine treatment, and syndrome differentiation and treatment). A systematically trained resident physician was present to evaluate the clinical ability of the students according to the modified Mini-CEX scale and gave the student real-time feedback.
5. Calculate scores of each field of Mini-CEX of the three groups.
