# Supplementary material for: Faculty standardized patients versus traditional teaching method to improve clinical competence among traditional Chinese medicine students: a prospective randomized controlled trial
Source: BMC Med Educ. 2024 Jul 24;24:793. doi: 10.1186/s12909-024-05779-3 (PMC11267817; doi:10.1186/s12909-024-05779-3)
Supplement: Supplementary file 4 — Supplement 4: eTable 1: Modified Mini-CEX. eTable 2: The Scoring Details of the Offline Clinical Skill Test [file 12909_2024_5779_MOESM4_ESM.docx]

**Modified Mini-CEX**

| **Domain** | **Content** | **Score** |
| --- | --- | --- |
| Medical interview ability | Proper addressing of patients and comfortable self introduction | 1 |
|  | The priority of the inquiry is clear, and the patient's chief complaint can be accurately summarized | 2 |
|  | The characteristics of the main clinical symptoms were inquired in detail (for example, the patients with pain as the main complaint should be inquired about their pain location, nature, duration, attack frequency, aggravating and relieving factors, etc.) | 2 |
|  | The general conditions (including the patient's diet, stool, sleep, spirit, weight, etc.) were inquired in detail | 1 |
|  | Asked about the past history in detail | 1 |
|  | Asked about personal history in detail | 1 |
|  | Asked about the family history in detail | 1 |
| physical examination ability | Protect patient privacy | 1 |
|  | Inform the patient of the purpose of the physical examination | 1 |
|  | The articles required for physical examination are complete | 1 |
|  | Wash hands regularly before and after contacting patients | 1 |
|  | Assist the patient in taking the correct position | 1 |
|  | The physical examination should be standardized, with moderate strength, and pay attention to humanistic care (such as covering the stethoscope with both hands) | 2 |
|  | The physical examination sequence is reasonable, and the patient's position is not changed repeatedly | 1 |
|  | The physical examination system is comprehensive, and no inspection items are omitted (such as tongue examination and pulse examination in TCM) | 1 |
| clinical judgment ability | Correct diagnosis by western medicine | 1 |
|  | Correct differential diagnosis by western medicine | 2 |
|  | Correct differentiation of diseases in TCM | 1 |
|  | Correct syndrome differentiation of TCM | 1 |
|  | Correct differential diagnosis of TCM | 2 |
|  | Correct analysis of TCM syndrome differentiation | 2 |
| disease treatment ability | The principle of western medicine treatment is accurate | 1 |
|  | Accurately arrange further auxiliary inspections | 1 |
|  | Correct treatment of TCM | 2 |
|  | Correct TCM Prescription | 1 |
|  | Explain the decoction and administration of TCM, precautions and possible body reactions (including efficacy and side effects) after taking the medicine to patients | 2 |
|  | Correctly analyze the TCM prescriptions | 2 |
| comprehensive ability | Ability to accurately judge the severity of illness | 3 |
|  | Ability to deal with emergencies | 3 |
|  | Complete the assessment within the specified time | 2 |
|  | Overall efficiency | 1 |

| **The Scoring Details of the Offline Clinical Skill Test.** | | |
| --- | --- | --- |
| **Item** | **criteria** | **Score (point)** |
| Application of TCM skills | Self-introduction | 4 |
|  | Chief complaint | 8 |
|  | Current medical history | 30 |
|  | Past medical history | 12 |
|  | Personal medical history | 12 |
|  | Family medical history | 8 |
|  | Physical examination, and the four TCM diagnostic methods | 16 |
|  | Summarization | 10 |
| Written Medical Records | General information | 3 |
|  | Chief complaint | 5 |
|  | Present medical history | 30 |
|  | Past medical history | 10 |
|  | Personal medical history | 10 |
|  | Family medical history | 6 |
|  | Physical examination | 20 |
|  | The four TCM examinations | 16 |
| TCM Syndrome Differentiation and Therapeutic Regimen | TCM diagnosis | 6 |
|  | TCM diagnostic basis | 6 |
|  | Western medicine diagnosis | 6 |
|  | Western medicine diagnostic basis | 14 |
|  | TCM syndrome type | 10 |
|  | TCM syndrome analysis | 24 |
|  | TCM treatment methods | 8 |
|  | Herbal prescription | 8 |
|  | Composition, administration method and specific dosage of herbal medicine | 14 |
|  | Medical advice | 4 |
