# Supplementary material for: Faculty standardized patients versus traditional teaching method to improve clinical competence among traditional Chinese medicine students: a prospective randomized controlled trial
Source: BMC Med Educ. 2024 Jul 24;24:793. doi: 10.1186/s12909-024-05779-3 (PMC11267817; doi:10.1186/s12909-024-05779-3)
Supplement: Supplementary file 5 — Supplement 5: The Cost Comparison Between FSP-TCM and OSP-TCM [file 12909_2024_5779_MOESM5_ESM.docx]

| **Table 5 The cost comparison between FSP-TCM and OSP-TCM** | | |
| --- | --- | --- |
| Items | FSP-TCM | OSP-TCM |
| Training expense  (One time/per person) | ¥2500.00 ($345.00)$\times$10 | ¥5000.00 ($690.00) $\times$10 |
|  |  |  |
| Qualification authentication  (One time/per person) | ¥200.00 ($27.60) $\times$10 | ¥200.00 ($27.60) $\times$10 |
|  |  |  |
| Course fee  (A credit hour/per person) | ¥50.00 ($6.90)$\times$10$\times$36 | ¥120.00 ($16.56) $\times$10$\times$36 |
|  |  |  |
| Traffic allowance  (One time/per person) | None | ¥50.00 ($6.90) $\times$12$\times$10 |
|  |  |  |
| Retraining expense  (One time biennially/per person) | ¥500.00 ($69.00) $\times$10 | ¥1000.00 ($138.00) $\times$10 |
|  |  |  |
| Re-qualification authentication  (One time biennially/per person) | ¥200.00 ($27.60) $\times$10 | ¥200.00 ($27.60) $\times$10 |
| Medical examination  (One time biennially/per person) | None | ¥1000.00 ($138.00) $\times$10 |
|  |  |  |
| Psychological assessment  (One time biennially/per person) | ¥300.00 ($41.40) $\times$10 | ¥300.00 ($41.40) $\times$10 |
|  |  |  |
| Total | ¥55000.00($7590.00） | ¥126200.00($17415.60） |

Note: ¥, RMB; $, Dollar; FSP-TCM, Faculty SP for Traditional Chinese medicine; OSP-TCM, Occupational SP for Traditional Chinese medicine;
